# AI-Based Pipeline for Classifying Pediatric Medulloblastoma Using Histopathological and Textural Images

**DOI:** 10.3390/life12020232

**Published:** 2022-02-03

**Authors:** Omneya Attallah, Shaza Zaghlool

**Affiliations:** 1 Department of Electronics and Communications Engineering, College of Engineering and Technology, Arab Academy for Science, Technology and Maritime Transport, Alexandria P.O. Box 1029, Egypt; 2 Bioinformatics Core, Weill Cornell Medical College in Qatar, Education City, Doha P.O. Box 24144, Qatar

**Keywords:** pediatric medulloblastoma subtypes, brain tumors, artificial intelligence, classification, deep learning, feature extraction, convolutional neural networks (CNN), texture analysis

## Abstract

Pediatric medulloblastomas (MBs) are the most common type of malignant brain tumors in children. They are among the most aggressive types of tumors due to their potential for metastasis. Although this disease was initially considered a single disease, pediatric MBs can be considerably heterogeneous. Current MB classification schemes are heavily reliant on histopathology. However, the classification of MB from histopathological images is a manual process that is expensive, time-consuming, and prone to error. Previous studies have classified MB subtypes using a single feature extraction method that was based on either deep learning or textural analysis. Here, we combine textural analysis with deep learning techniques to improve subtype identification using histopathological images from two medical centers. Three state-of-the-art deep learning models were trained with textural images created from two texture analysis methods in addition to the original histopathological images, enabling the proposed pipeline to benefit from both the spatial and textural information of the images. Using a relatively small number of features, we show that our automated pipeline can yield an increase in the accuracy of classification of pediatric MB compared with previously reported methods. A refined classification of pediatric MB subgroups may provide a powerful tool for individualized therapies and identification of children with increased risk of complications.

## 1. Introduction

Pediatric medulloblastoma (MB) is one of the most life-threatening central nervous system (CNS) tumors affecting children [1,2]. MB is a small blue cell malignancy of the cerebellum, which eventually progresses to other brain regions [3]. These tumors account for almost 25% of all pediatric tumors [4] and are the leading cause of cancer-related death in children below 15–16 years of age [5,6]. Nearly 20% of CNS tumors in children are in some form of MBs [7,8]. There are four consensus subgroups of MB, each characterized by distinct clinical and molecular features that are now widely recognized, namely, wingless activated (WNT), Sonic hedgehog activated (SHH), Group 3, and Group 4 [9]. Being the most common type of brain cancer leading to death in children, precise and timely detection of such tumors is vital in terms of planning treatment regimens and improving disease progression and outcomes.

The first-line imaging modality used in the diagnosis of suspected pediatric brain tumors of the CNS is conventional magnetic resonance imaging (MRI) and resting-state functional MRI [10,11,12]. Although the resting-state functional MRI offers a variety of data regarding qualitative changes, this comprehensive knowledge does not automatically deliver a profound understanding of the way to utilize this attained information in contrary engineering [12]. Several studies [13,14,15,16] have used MRI to classify brain tumors. Despite current advances in MRI that provide remarkable structural detail, classification of MB subgroups can still be quite challenging in terms of identifying tumor type, aggressiveness, and metastatic potential [17]. MRI provides a non-invasive high-throughput output that can be used to extract quantitative image features through methods such as texture analysis (TA) [18]. However, there are challenges in using MRI for diagnosing pediatric MB subtypes [19], such as the fact that many brain tumor types in MRI scans are frequently indistinguishable in terms of pattern and visual appearance [20]. Since using standard MRI for diagnosis might result in an imprecise decision [21], different imaging methods are favored in identifying MB and its subtypes [19]. Currently, the traditional method for diagnosis of MB is through manual histopathology analysis of tumor samples [22]. This process can be inefficient, expensive, and highly biased. Usage of computer-based algorithms has enabled assessment of both semantic image features (i.e., size, shape, intensity, or contrast) and diagnostic features (i.e., histograms, textures, or wavelets).

Computational methods based on machine and deep learning have been used extensively for classification and diagnosis across various cancer and human disorders or diseases [23,24,25,26,27,28]. However, very few studies have used automated image analysis to study the four subgroups of MB [29,30]. The main roadblock in the classification of pediatric MB subgroups by applying computational methods to histopathological images lies in the limited availability of datasets containing the four subgroups. Most previous studies carried out classification on privately acquired datasets containing only anaplastic and non-anaplastic MB. Studies used various methods including Haar and MR8 wavelets, Haralick Laws texture features, random forest, and k-nearest neighbor (k-NN) classifiers, for binary classification, which yielded accuracy between 80% and 91% [31,32]. Others used various feature extraction techniques including the bag of features histogram, visual latent semantic features, sparse auto-encoders, topographic independent component analysis (TICA), and 3-layered convolution neural networks (CNN) [33,34]. The highest accuracy (97%) was obtained using a 2-layer CNN using the TICA method combined with wavelet transform [35].

Most previous techniques for MB subgroup classification relied on a single feature extraction method. Feature extraction methods that were used in pediatric MB subgroup classification included the gray level co-occurrence matrix (GLCM) texture-based method [36], local energy pattern extraction [37], deep learning, or other texture analysis methods [38,39]. These methods are sensitive to parameter choice, and thus, prone to error. For example, the GLCM method can be very sensitive to the choice of distance metric, which must take into consideration various textures and regional spatial dependence. Image quality also greatly impacts the feature extraction process and the performance of classification. Although the local energy pattern feature extraction method and texture-based feature extraction methods are not dependent on the imaging settings, they may still not capture the discriminative dataset characteristics.

Thus far, most studies have focused only on the binary classification of pediatric MB. Few studies [40] have attempted to classify the four MB subgroups using machine and deep learning techniques applied to histopathological images. In those studies, the images were first segmented using k-means clustering prior to morphological and color feature extraction, including GLCM features, histogram of oriented gradients (HOG), Tamura, local binary pattern (LBP) features, and gray level run matrix (GLRM) features. Color features may be impacted by light source and occlusion artifacts [41,42]. Principal component analysis (PCA), as well as multivariate analysis of variance (MANOVA) [32], were used for feature dimensionality reduction. Das et al. [43] also experimented with various combinations of feature sets and fused four features to obtain a classification accuracy of 96.7% using PCA as a feature reduction method and a support vector machine (SVM) classifier. Only texture-based features were used in that study. Later, Das et al. [44] used pre-trained convolutional neural networks (CNNs), specifically AlexNet and VGG-16. Soft-max and SVM classifiers were used to obtain almost 94% classification accuracy. This method did not combine different deep learning approaches. Taking it further, Attallah et al. [45] developed a framework based on three CNNs and incorporated PCA, with discrete cosine transforms, applied to deep features for input to four different classifiers. This approach combines texture-based feature extraction with ten deep learning (DL) methods. To reduce the feature dimension, feature fusion was carried out using the discrete wavelet transform (DWT).

In this study, we propose an automated pipeline based on artificial intelligence (AI) methods to classify the four subtypes of pediatric MB brain tumors as defined by the WHO. Previous studies used a single feature extraction method before classification, such as deep learning or textural analysis, and only used the original images to perform classification. Here, we not only used the original images but converted these images to textural images using two well-known texture analysis methods and then fed three deep learning models with these images. We also trained these three deep learning models with the original images and extracted deep features from the models trained with textural images and original images. Finally, we integrated multiple deep features obtained from such deep learning models to combine the benefits of different strategies, spatial, and textural information from the original and textural images. Our proposed method improves the precision in the identification of pediatric MB subclasses and decreases the risk of misidentification, thus aiding in patient diagnosis and tailoring of treatment plans.

## 2. Materials and Methods

### 2.1. Convolutional Neural Networks

In this study, we use three state-of-the-art Convolutional neural networks (CNN). CNNs are a well-known class of deep learning techniques that are commonly used for analyzing medical images and performing classification or diagnosis [46]. The main structure of CNN is the perceptron model. The main strength of CNNs over conventional artificial neural networks lies in their ability to automatically extract features from an image, making CNNs a hot research topic, particularly in medical image processing field [47,48]. These networks have a great capacity to employ images directly for diagnosis, eliminating the excessive processing steps that are usually required in conventional machine learning techniques such as preprocessing, segmentation, and feature extraction [48,49]. Moreover, CNNs can decrease the complexity of classification models by making use of both the local and global information of a medical scan by performing vigorous rotation, translation, and scaling. The three main layers of any CNN consist of the convolutional, pooling, and fully connected (FC) layers. Within the convolutional layers, convolution is carried out between segments of an image and a filter of small size. Following that, a feature map is produced containing the spatial information of the pixels in each segment of an image. Since the generated feature maps are large, the pooling layer then serves a major role in diminishing the huge dimension of such features by downsampling. Finally, the FC layer gathers inputs from the previous layers and generates class scores. In this study, we use three CNNs specifically: the ResNet-101, Inception, and InceptionResNet models.

ResNet is a popular CNN commonly used in medical image analysis. The main component of ResNet depends on the residual block launched by He et al. [50]. This residual block finds short routes within the convolutional layers enabling the CNN to skip certain convolutional layers. During training, the CNN chooses between two routes to follow; either it performs a number of operations on the input, or ignores that route. These shortcuts speed up parameter updating and counteract the gradient vanishing problem that is bound to occur with the backpropagation algorithm. In this study, we used ResNet-101, which consists of 100 convolutional layers and one FC layer.

Inception is another well-known CNN introduced in 2016 by [51]. This CNN is based on GoogleNet [52]; however, it has much lower memory requirements and computational requirements. The key element in the Inception model is the integration of multiple filters with distinct dimensions into one new filter. This new filter reduces the number of parameters, thus reducing training time [51]. To maximize data flow, the Inception block takes into consideration the depth and width of the layers throughout the CNN training phase. The Inception CNN is 48 layers deep.

The InceptionResNet is a combination of ResNet and Inception. It simply presents residual shortcuts within the Inception block [53] so that the new filter created in the Inception block is pooled in the residual shortcuts. The InceptionResNet is capable of considerably improving the training performance and time compared to the Inception and ResNet models. This CNN is 164 layers deep.

### 2.2. Texture Analysis Methods

Texture analysis is a well-known method that is commonly used to analyze medical images. The textural analysis consists of several computation steps applied to medical images [54]. The most common textural analysis methods include the gray-level covariance matrix (GLCM) and gray level run length matrix (GLRM), and they are widely used in medical applications [55,56,57,58,59,60]. These methods generally yield sufficient performance, especially when combined [58].

The GLCM approach is a second-order histogram method that relies on the grey level distribution between pairs of pixels. GLCM computes the common frequencies of the whole pairwise mixtures of the grey level composition of every pixel in the left hemisphere (at different angles), which is taken as a reference pixel with each of the opposite pixels in the right hemisphere. Accordingly, multiple covariance matrices are generated corresponding to each pairwise combination of pixels. Afterwards, every covariance matrix is normalized by the total number of its components to determine the covariance relative frequency among the gray levels of mutual pixels [61]. In this study, we have used the traditional GLCM textural features approach, although other techniques such as the doughnut GLCM [62], GLCM based on Haralick features [58], WPT-GLCM, WPT-LBP-GLCM, and WPT-Gabor-GLCM (WPT: wavelet packet transform, LBP: local binary patterns) [63] have been deployed by other studies.

The GLRM method extracts high-order statistical textural features, where a gray level run represents a line of pixels with the same intensity all in a certain direction [36]. For each medical image of size N × M, GLCM calculates the number of gray levels G, which is a string containing the pairwise pixels having a similar gray-level intensity in a particular direction and the longest run L, respectively. The GLRM is a bi-dimensional matrix of (G × L) elements, in which every element *Q* (*m*, *n*), provides the number of occurrences of the run, which has a size ***n*** of gray level ***m*** in a certain path θ [64].

### 2.3. Data Collection

The medical images used in this study were collected at two medical centers, the Guwahati Neurological Research Center (GNRC) and the Guwahati Medical College and Hospital (GMCH), as described previously by Das et al. [65]. The dataset consists of pediatric MB tumor images for children with age <15 years. The extracted tissues were stained with hematoxylin and eosin (HE) by a local medical pathologist at Ayursundra Pvt. Ltd. After the regions of interest were determined by a specialist, the images were amplified by a factor of 10× using a Leica 1CC50 HD microscope and saved in JPEG format. Each MB tumor image in the dataset was assigned a label that corresponded to one of the four MB subclasses. A total of 154 images corresponding to 59, 42, 30, and 23 classic, desmoplastic, large cell, and nodule MB, respectively, were available for analysis.

### 2.4. Proposed Pipeline

**Figure 1:**
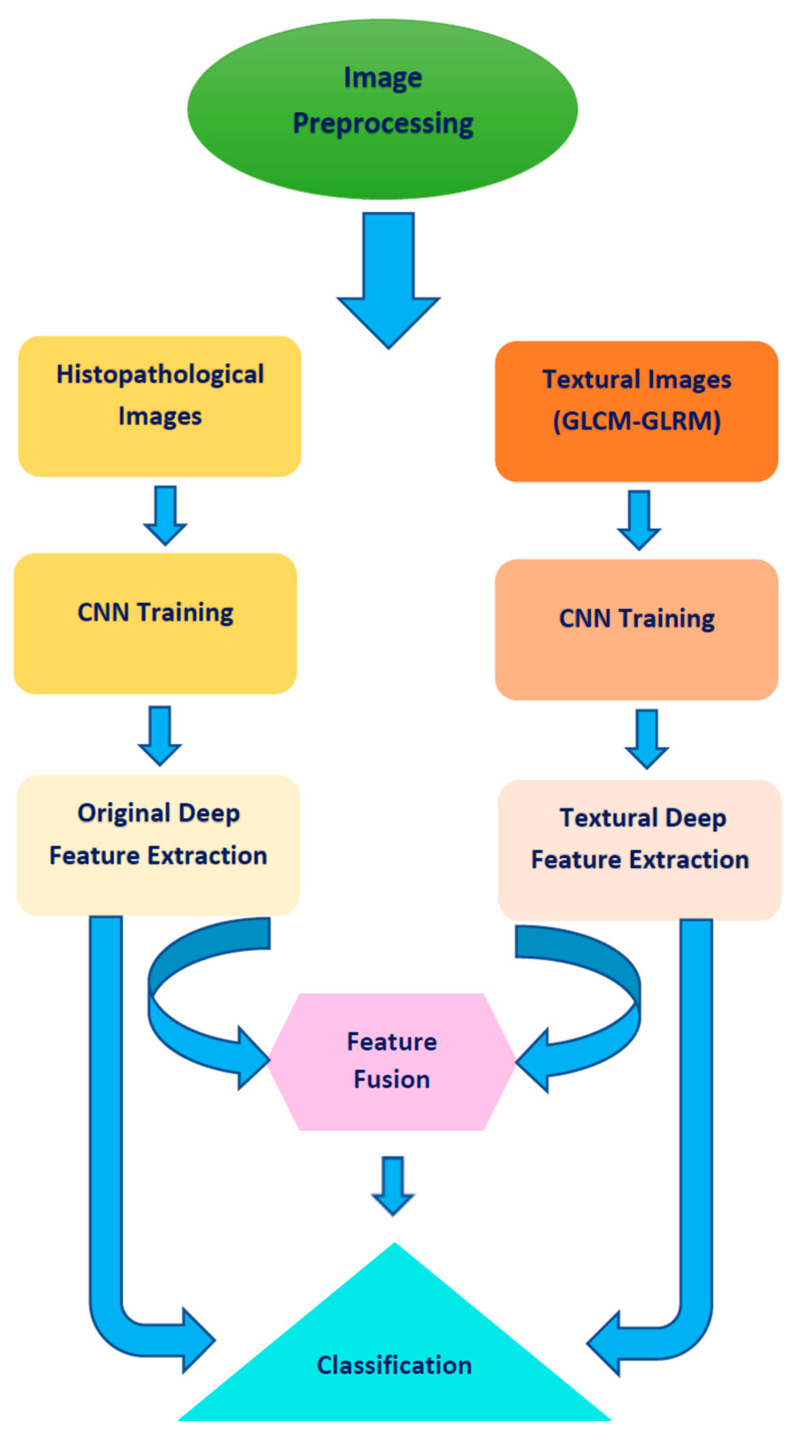
Steps of the proposed pipeline.

We propose a pipeline based on multiple deep learning methods to classify MB subclasses. The pipeline consists of four steps including image preprocessing, textural image generation and CNN training, feature extraction and fusion, and classification. In the first step, the images are resized and augmented. Next, textural analysis is used to analyze the original histopathological images and generate textural images. Both the original histopathological images and textural images are then used to train three CNNs individually. Afterwards, deep features are extracted from these CNNs, which were either trained with the original images or the textural images. Then, these features are used independently to train three machine learning classifiers. Finally, these features are concatenated and used to train the three machine learning classifiers. The steps of the proposed pipeline are shown in Figure 1.

#### 2.4.1. Image Preprocessing

To begin with, we resized the images according to the size of the input layer for the different CNN architectures used in this study, which are 224 × 224 × 3 for ResNet-101, and 229 × 229 × 3 for Inception and InceptionResNet. To optimize the quality of our training dataset, we then used data augmentation to increase the number of available images, as described by [66]. The data augmentation methods that we used included translation (−30,30), scaling (0.9, 1.1), flipping in x and y directions, and shearing (0, 45) in the x and y directions, as done previously in [67,68].

#### 2.4.2. Textural Image Generation and CNN Training

**Figure 2:**
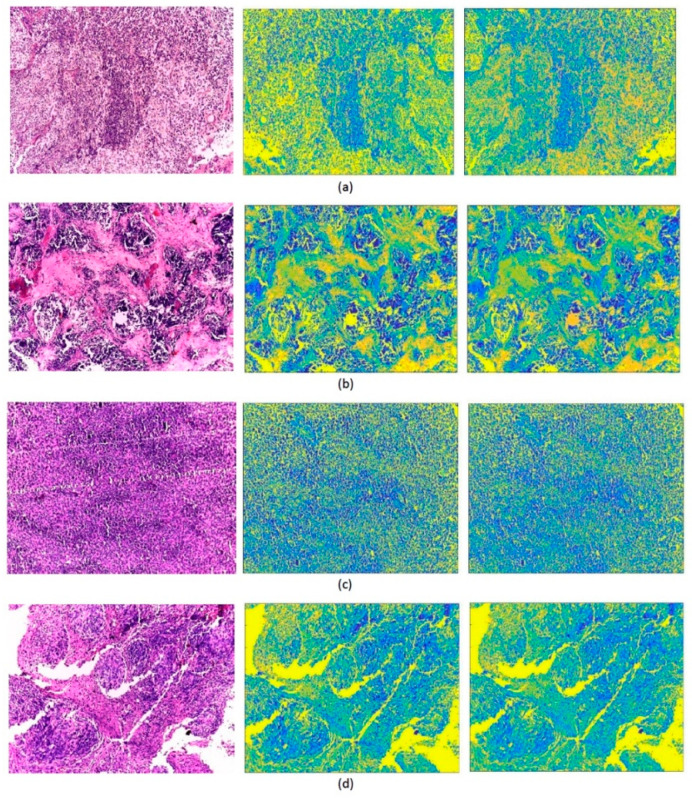
Sample images for the four pediatric MB subclasses: (**a**) classic, (**b**) desmoplastic, (**c**) large cell, and (**d**) nodular. The left column consists of the original images, while the GLCM and GLRM textural images are shown in the middle and right columns, respectively.

To generate textural images, we applied the GLCM and GLRM texture analysis methods to the original images. We then generated heatmaps from the output features of the GLCM and GLRM methods, which we converted into images. For the GLCM and GLRM methods, we applied four orientations (0, 45, 90, and 135), respectively, and 8 gray levels. We then constructed three pre-trained CNNs previously trained on the ImageNet dataset using transfer learning (TL). TL [69] uses an existing CNN architecture that was designed for natural image datasets with their pre-trained weights, and then tweaks the model on medical imaging data. It is frequently used in the medical field, since acquiring large labeled image datasets such as the ImageNet dataset is very difficult [70]. The output layers of ResNet-101, Inception, and InceptionResNet CNNs were set to four, which corresponds to the number of MB subtypes instead of the original 1000 used in the ImageNet dataset. In addition, we set some parameters such as the number of epochs, validation frequency, mini-batch size, and the initial learning rate to 200, 26, 4, 0.0003, respectively. The three CNNs were then trained individually with the GLCM and GLRM images. In parallel, each of the three CNNs was trained with the original images. Figure 2 shows samples of the original images for the four pediatric MB subclasses along with their GLCM and GLRM images.

### 2.5. Feature Extraction and Fusion

In this step, TL is again used with three pre-trained CNNs, including ResNet-101, Inception, and InceptionResNet, to allow each CNN to be used as a feature extractor. We extracted deep spatial features and deep textural features. The deep spatial features were extracted from the three CNNs trained using the original images. These features were obtained from the last fully connected layer of each CNN. The deep textural features were extracted from the fully connected layer of each CNN separately, using TL. Fusion was then carried out in two steps. In the first step, the deep textural features were fused to compare their performance to the spatial deep features. In the second step, both types of textural deep features (GLCM + GLRM) were combined with the deep spatial features to determine whether fusing spatial and textural features enhances the diagnostic accuracy. The length of the individual features sets (spatial or textural) was 4, while the length of the combined features in the first and second steps were 8 and 12, respectively.

### 2.6. Classification

We carried out classification using three classifiers, support vector machines (SVM), linear discriminant analysis (LDA), quadratic discriminant analysis (QDA), naïve bayes (NB), K-nearest neighbor (K-NN), and random forest (RF) classifiers. SVMs use supervised learning to robustly make non-linear classification using a kernel, which implicitly maps data into high-dimension feature spaces [71]. LDA is a method that is a generalization of Fisher’s linear discriminant and searches for a linear combination of features that characterize or separate different classes. QDA is similar to LDA in terms of its assumption that the measurements from each class are normally distributed. However, in QDA, there is no assumption that regards the covariance of each class as identical [72].

Using the three classifiers mentioned above, we further performed classification using three different approaches. In the first approach, we used deep spatial features to train the three classifiers individually. In the second approach, we used the textural features of GLCM and GLRM separately to train the three classifiers individually. In the third approach, we fused the deep textural features extracted from both CNNs to train the three classifiers. The fused deep textural features were then combined with the spatial deep features extracted from each CNN trained with the original MB images. The third approach allowed us to evaluate whether fusing different types of features improved the performance of the proposed model.

## 3. Performance Evaluation Metrics

The performance of the proposed pipeline was evaluated using several evaluation metrics including accuracy, precision, sensitivity, F-1 score, specificity, Mathew correlation coefficient (MCC), receiving operating characteristic curve (ROC), and the area and the ROC curve (AUC). Equations (1)–(6) were used to compute these metrics.

(1) $$Accuracy=\frac{TP+TN}{TN+FP+FN+TP}$$

(2) $$Sensitivity=\frac{TP}{TP+FN}$$

(3) $$Specificity=\frac{TN}{TN+FP}$$

(4) $$Precision=\frac{TP}{TP+FP}$$

(5) $$F1-Score=\frac{2\times TP}{2\times TP+FP+FN}$$

(6) $$MCC=\frac{TP\times TN-FP\times FN}{\sqrt{\left(TP+TN\right)\left(TP+FN\right)\left(TN+FP\right)\left(TN+FN\right)}}$$

where:

**True Positives (TP):** is the number of scans where the model correctly predicts the positive class.
**False Positives (FP):** is the number of scans where the model incorrectly predicts the positive class.
**True Negatives (TN):** is the number of scans where the model correctly predicts the negative class.
**False Negatives (FN):** is the number of scans where the model incorrectly predicts the negative class.

## 4. Results and Discussion

### 4.1. First Fusion Step

Following the steps presented in Figure 1, we classified the four subtypes of pediatric MB brain tumors using alternative approaches and compared the performance of each approach. We trained six different classifiers (LDA, QDA, SVM, NB, KNN, and RF classifiers) with deep features extracted from three fully connected layers of three CNN architectures (ResNet-101, Inception, and InceptionResNet). In the first fusion stage, these CNNs were constructed using the original images and textural images (GLCM and GLRM). Then deep textural features obtained from the CNNs trained with textural (GLCM and GLRM) images were concatenated. The performance of the three classifiers constructed with the fused textural features was compared to the performance of the same classifiers trained with each type of deep textural features individually. Furthermore, the performance of the three classifiers trained with the fused deep textural features was compared to the performance of the same classifiers trained with an individual textural feature extraction method. The results of these comparisons are shown in Table 1. The classification accuracies for the six classifiers trained with the fused deep textural features were higher than those obtained by the same classifiers when trained with a single type of deep textural features (GLCM or GLRM). The accuracies for the LDA, QDA, SVM, NB, KNN, and RF classifiers were 98.34%, 98.7%, 97.04%, 98.7%, 98.1%, and 96.7%, respectively, for ResNet-101 CNN, which were higher than accuracies obtained when these classifiers were trained with individual deep textural features. Our results provide confirmation that fusing textural features enhances the classification accuracy.

Furthermore, the classification accuracies were generally higher for the original images compared to the individual textural images (GLCM or GLRM). The GLCM textural images appeared to yield the lowest classification accuracies. However, when the textural images were combined (GLCM and GLRM), we found a comparable classification accuracy to using the original images. We observed a similar trend for all three classifiers, ranging from 96.8% to over 99.4% for the original images and from 96.64% to over 98% for the combined textural images. See Table 1 for details.

### 4.2. Second Fusion Step

**Figure 3:**
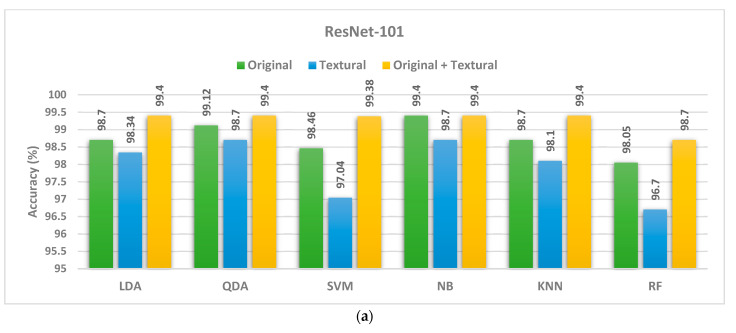

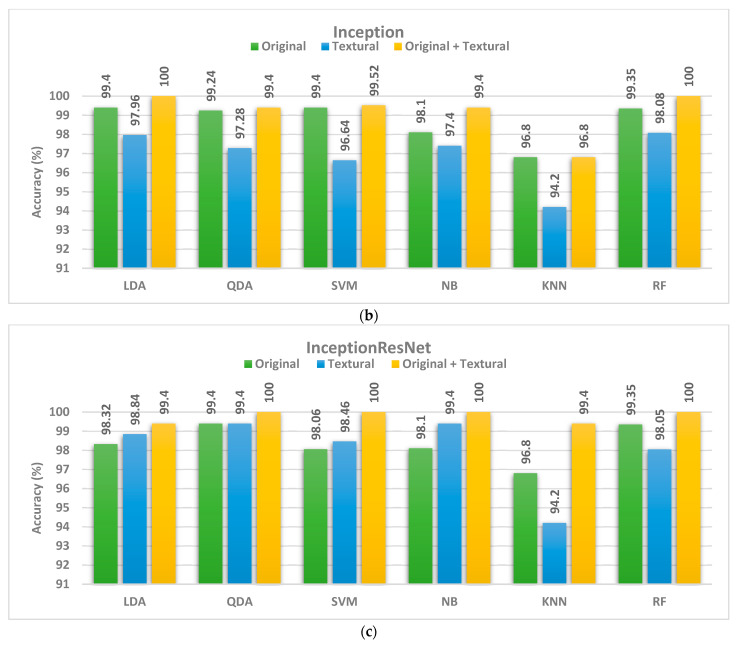
Comparison of classification accuracy for three classifiers (LDA, QDA, and Q-SVM) using various CNNs structures, i.e., CNNs constructed using the original image, the textural images (GLCM and GLRM), and all images (Original + GLCM + GLRM). The classification accuracy was highest when combining the original and textural images for all three tested CNNs ((**a**) ResNet-101, (**b**) Inception, and (**c**) InceptionResNet).

In the second fusion step, we tested whether adding textural images to the original images can further improve the classification accuracy. Indeed, we observed an additional increment in the classification accuracy when comparing the usage of spatial deep features obtained from CNNs trained with original image features alone to the original and textural image features combined. This improvement was observed across all six classifiers and all three CNNs. Optimal classification accuracy of 100% was achieved using the LDA or RF classifier with the Inception CNN, and similarly using the QDA, SVM, NB, or RF classifiers with the InceptionResNet CNNs scheme (Figure 3). Following the same trend, accuracies of 99.38% and 99.52 were obtained using the SVM classifier trained with the combined features (original + textural images) for the ResNet-101 and Inception models, respectively. An accuracy of 99.4% was obtained using the LDA classifier trained with the combined features (original + textural images) obtained from the ResNet-101 and Inception models. Finally, an accuracy of 99.4% was obtained using the QDA classifier with the combined features (original + textural images) for the Inception model. These accuracies provide validation that merging both spatial and textural information enhances classification performance.

**Figure 4:**
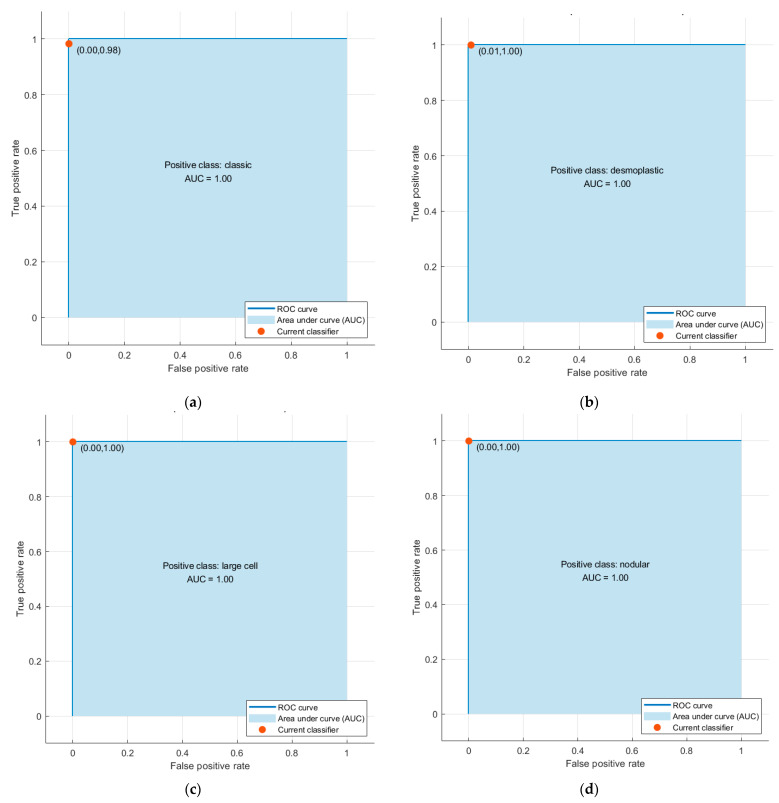
The ROC curves and the AUCs obtained using the QDA classifier trained with combined features of the ResNet-101 model where the positive class is (**a**) classic, (**b**) desmoplastic, (**c**) large cell, and (**d**) nodular.

**Figure 5:**
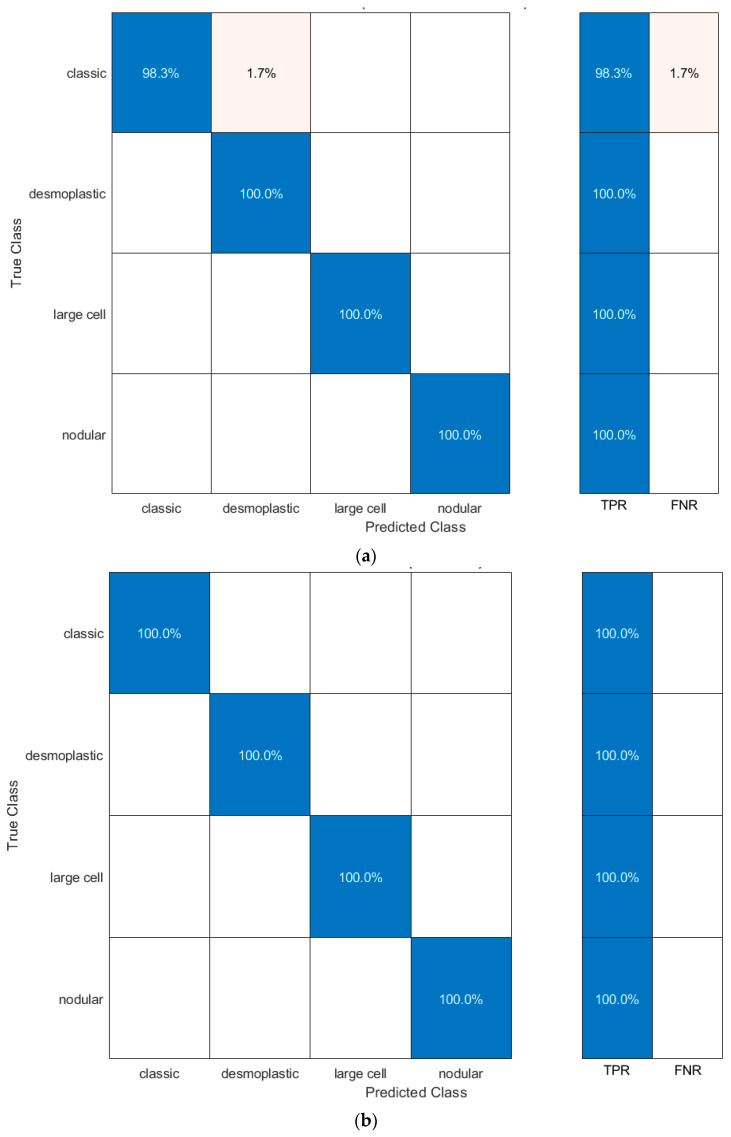
Confusion matrix for the QDA classifier: (**a**) trained with combined features of the ResNet-101 model; (**b**) trained with combined features of the InceptionResNet model.

The performance metrics for the LDA classifier trained with the fused features (original + textural images) are presented in Table 2. These CNNs were trained using the original images and textural images (GLCM and GLRM). The results in Table 3 show the performance metrics for the LDA classifier trained with combined features (spatial information from original images and textural information from GLCM and GLRM images). The ROC curves and the AUCs obtained using the QDA classifier trained with the combined features of the ResNet-101 model are also presented in Figure 4. All AUCs were equal to 1. Finally, the confusion matrix for the QDA classifier trained with combined features is presented in Figure 5.

### 4.3. Comparision with Other Methods and Studies

**Figure 6:**
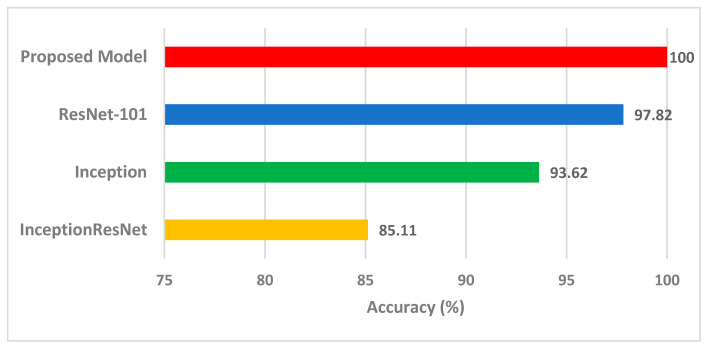
Performance of the proposed pipeline compared to state-of-the-art end-to-end deep learning classification of the three CNN models used.

The highest accuracy obtained using the proposed pipeline was compared with the state-of-the-art end-to-end deep learning classification of the three CNN models used to construct the proposed pipeline (Figure 6). The accuracy of the proposed pipeline is significantly higher than that obtained with InceptionResNet, Inception, or ResNet-101 CNNs, confirming that the proposed pipeline is superior to end-to-end deep learning classification.

Finally, we compared our proposed model with previous studies. We showed that combining the original histopathological images with textural images yields an improvement in the overall classification accuracy as well as other performance metrics (Table 3). Furthermore, the number of final features obtained using the final model is 12, which is much lower than that obtained in the related studies. The results of the proposed pipeline show its strength and superiority over all other methods of the literature based on the same dataset.

## 5. Conclusions

We propose an automated pipeline based on various deep learning methods to aid in the classification of the heterogeneous pediatric MB subtypes. By combining information from textural images with the original histopathological images, we improved the classification accuracy reaching an outstanding classification accuracy of 100%. Our study presents an improvement to current methods where only a single feature extraction method and/or a single classifier are used. This enhancement in the classification of pediatric MB subgroups may aid clinicians in MB subtype diagnosis, identification of children with increased risk of complications, and design of individualized therapies.

## Figures and Tables

**Table 1:** Classification accuracies (%) are compared for different classifiers (LDA, QDA, and Q-SVM) trained with deep features extracted from different CNN fully connected layers (ResNet-101, Inception, and InceptionResNet). The CNNs were constructed using the original image, the individual textural images (GLCM and GLRM), and both textural images (GLCM + GLRM).

	Original Images	GLCM	GLRM	GLCM+ GLRM
	**ResNet-101**			
LDA	98.70	94.68	95.98	98.34
QDA	99.12	94.18	97.16	98.7
SVM	98.46	92.88	96.26	97.04
NB	99.40	96.80	97.40	98.70
KNN	98.70	95.50	96.10	98.10
RF	98.05	94.80	96.10	96.7
	**Inception**			
LDA	99.40	92.60	97.82	97.96
QDA	99.24	92.86	96.64	97.28
SVM	99.40	93.28	96.38	96.64
NB	98.1	92.22	96.88	97.40
KNN	96.80	89.60	93.50	94.20
RF	99.35	88.96	95.45	98.05
	**InceptionResNet**			
LDA	98.32	96.38	98.58	98.84
QDA	99.40	95.48	96.78	99.40
SVM	98.06	95.74	97.44	98.46
NB	98.1	96.1	98.70	99.4
KNN	96.1	96.1	97.4	97.40
RF	96.1	94.80	98.70	98.7

**Table 2:** Performance metrics for an LDA classifier that was trained using the fused features extracted from the last fully connected layer of the ResNet-101, Inception, and InceptionResNet CNNs. These CNNs were trained using the original images and textural (GLCM and GLRM) images.

Classifier	Sensitivity	Specificity	Precision	F1-Score	MCC
ResNet-101	0.9958	0.9977	0.9942	0.9974	0.9925
Inception	1	1	1	1	1
InceptionResNet	0.9958	0.9981	0.9895	0.9918	0.9904

**Table 3:** Comparison of the proposed model with previous studies. Our proposed model outperforms all previous related work with respect to various performance metrics.

Model Description	Precision (%)	Accuracy (%)	Sensitivity (%)	Specificity (%)	Number of Features	Reference
Proposed Model	100	100	100	100	12	This study
Shape + Color features; PCA; SVM	-	84.9	-	-	19	[40]
HOG, GLCM, Tamura, LBP features; GRLN; MANOVA; SVM	66.6	65.2	72.0	-	34	[73]
AlexNet	-	79.3	-	-	-	[44]
VGG-16	-	65.4	-	-	-	[44]
AlexNet; SVM	-	93.2	-	-	4096	[44]
VGG-16; SVM	-	93.4	-	-	4096	[44]
GLCM, Tamura, LBP, GRLN features; SVM	91.3	91.3	91.3	97	83	[43]
GLCM, Tamura, LBP, GRLN features; PCA; SVM	-	96.7	-	-	20	[43]
MobileNet; DenseNet; ResNet merging using PCA; LDA classifier	99.6	99.4	99.5	99.6	95	[45]
Deep features from DenseNet-201, ShuffleNet; Relief-F; Bi-LSTM	98.1	98.1	98.1	99.3	448	[74]
Deep features from DenseNet-201, Inception, Resnet-50, Darknet-53, MobileNet, ShuffleNet, SqueezeNet, NasNetMobile; Relief-F; Bi-LSTM	99.4	99.4	99.8	99.4	739	[74]
FractalNet; GLCM, Tamura, LBP, GRLN; SVM	-	91.3	-	-	-	[30]

## Data Availability

The dataset analyzed can be downloaded from the IEEE Dataport https://ieee-dataport.org/open-access/childhood-medulloblastoma-microscopic-images. accessed on 14 November 2020.
